# CO _2_ elevation improves photosynthetic performance in progressive warming environment in white birch seedlings

**DOI:** 10.12688/f1000research.2-13.v1

**Published:** 2013-01-15

**Authors:** Shouren Zhang, Qing-Lai Dang

**Affiliations:** 1 State Key Laboratory of Vegetation and Environmental Change, Institute of Botany, The Chinese Academy of Sciences, Beijing, China; 2 Faculty of Natural Resources Management, Lakehead University, Ontario, Canada

## Abstract

White birch (Betula paperifera Mash) seedlings were exposed to progressively warming in greenhouses under ambient and elevated CO _2_ concentrations for 5 months to explore boreal tree species’ potential capacity to acclimate to global climate warming and CO _2_ elevation. In *situ* foliar gas exchange, in vivo carboxylation characteristics and chlorophyll fluorescence were measured at temperatures of 26 ^o^C and 37 ^o^C. Elevated CO _2_ significantly increased net photosynthetic rate (Pn) at both measurement temperatures, and Pn at 37 ^o^C was higher than that at 26 ^o^C under elevated CO _2_. Stomatal conductance (gs) was lower at 37 ^o^C than at 26 ^o^C, while transpiration rate (E) was higher at 37 ^o^C than that at 26 ^o^C. Elevated CO _2_ significantly increased instantaneous water-use efficiency (WUE) at both 26 ^o^C and 37 ^o^C, but WUE was markedly enhanced at 37 ^o^C under elevated CO _2_. The effect of temperature on maximal carboxylation rate (Vcmax), PAR-saturated electron transport rate (Jmax) and triose phosphate utilization (TPU) varied with CO _2,_ and the Vcmax and Jmax were significantly higher at 37 ^o^C than at 26 ^o^C under elevated CO _2_. However, there were no significant interactive effects of CO _2_ and temperature on TPU. The actual photochemical efficiency of PSII (DF/ Fm’), total photosynthetic linear electron transport rate through PSII (JT) and the partitioning of JT to carboxylation (Jc) were higher at 37 ^o^C than at 26 ^o^C under elevated CO _2_. Elevated CO _2_ significantly suppressed the partitioning of JT to oxygenation (Jo/JT). The data suggest that the CO _2_ elevation and progressive warming greatly enhanced photosynthesis in white birch seedlings in an interactive fashion.

## Introduction

Global climate warming and increases in atmospheric CO _2_ concentration are currently key topics for scientists, politicians and the general public alike ^1^. Such changes have been observed in the past 150 years and supported by modeling results for the longer term, *e.g.*, warming ocean water, shrinking mountain glaciers, retreating snow cover, and CO _2_ concentration dynamics in Arctic/Antarctic ice cores ^2– 6^. It is projected that the global temperatures will increase by an average of 3°C with a range of 2 to 4.5°C under the scenario of doubling atmospheric CO _2_ concentration by the end of the century ^2^. Global climate warming will likely have profound and diverse impacts on biological systems ^2, 4– 13^.

Increases in CO _2_ and temperature to a certain extent should have positive impact on photosynthesis and growth, as the current atmospheric CO _2_ concentration is below the saturation point for RuBisCO (Ribulose-1,5-bisphosphate carboxylase oxygenase) ^14^. Furthermore, higher CO _2_ concentrations suppress photorespiration and increase the partitioning of photosynthetic electron transport to carboxylation ^14^. However, the situation will become complicated if the temperature goes beyond plants’ ability to acclimate, or when the rate of temperature increase exceeds the pace of acclimation. In such cases, temperature and CO _2_ will have opposite effects on photosynthesis, i.e., the higher temperature induced increase in photorespiration may exceed the beneficial effect of CO _2_ elevation, resulting in a decline in net photosynthesis. Consequently, the direction and magnitude of change in net photosynthesis will be determined by the relative magnitudes of the two opposite effects ^15^. Kirschbaum ^16^ has conducted a theoretical analysis on the dependence of photosynthesis on temperature and CO _2_ concentration for C3 plants and found that at 35°C, photosynthesis at the ambient CO _2_ concentration reaches only 50% of the rate at saturating CO _2_ concentration, whereas the corresponding value at 5°C is 77%. Therefore, there is greater potential photosynthetic enhancement by CO _2_ elevations at higher temperatures. This theory has been supported by the results of a number of studies ^15, 17– 19^. Long ^20^ has suggested that the increase in atmospheric CO _2_ will not only increase photosynthetic rate, but also alter the photosynthetic response to temperature. Mooney *et al.* ^21^ indicate that the photosynthetic acclimation to elevated temperature and CO _2_ mainly involves changes in the heat stability of the thylakoids and RuBisCO activity. Hence, high temperature and CO _2_ elevations may have synergistic effect on photosynthesis and CO _2_ elevations may lead to improved acclimation to high temperatures. However, such interactions may vary with plant species ^22, 23^ and other environmental conditions. Variations in acclimation ability can change the interactions within and between species and the composition and functioning of plant communities under future climatic conditions. Furthermore, in most past studies, high temperature treatments are achieved in one step, which is in contrast with the gradual, progressive increases in temperature occurring in global climate changes.

**Figure 1.**
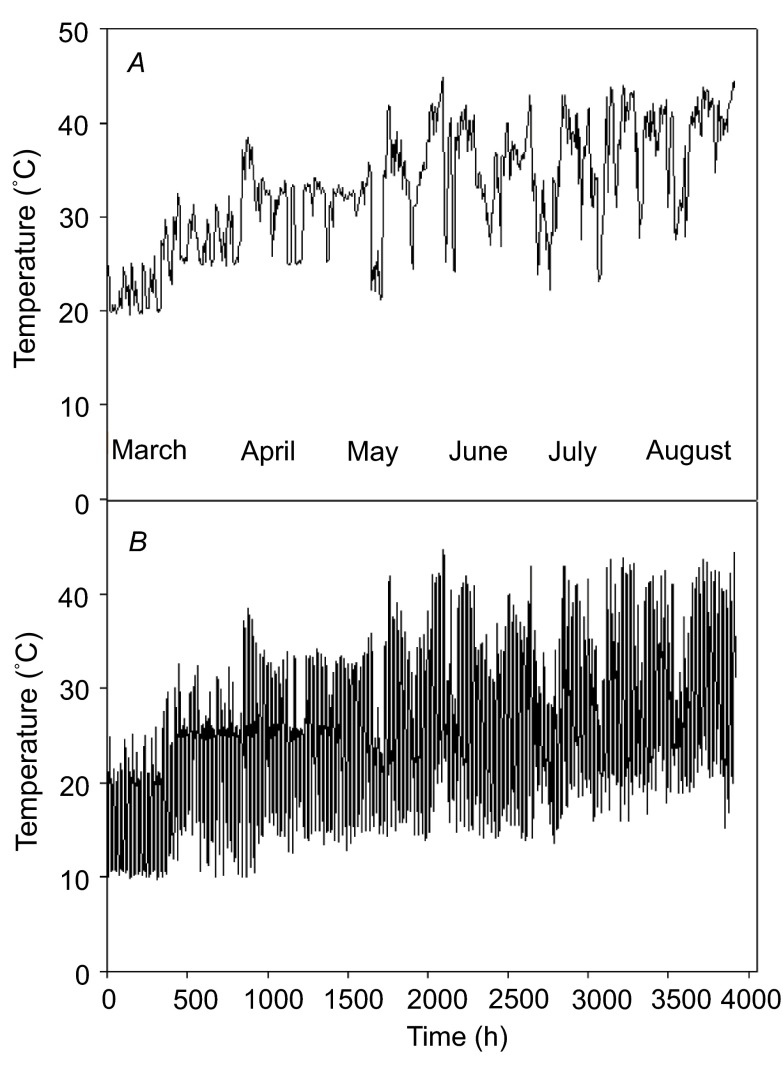
Time course of temperatures in the greenhouses during the experimental period (March 1 through August 15). ( **A**) Postmeridian (hour) pattern between 13:00 and 16:00; ( **B**) Diel (hour) pattern between 0:00 and 24:00.

The boreal forest is an important terrestrial ecosystem with a high carbon sequestration potential ^24^. As the global climate change accelerates, the boreal forest has been experiencing progressive increases in temperatures and CO _2_. The response of the boreal forests could have great impact on the global carbon balance ^25^. White birch is one of the most widely distributed tree species in the boreal forest. The growth conditions of white birch in northwest Ontario are characterized by a long cold winter and short summer. For example, the annual mean temperature in Thunder Bay region is 2.4°C while the January and July average temperatures are -14.9°C and 17.6°C (based on Environment Canada’s online weather records for the time period of January 1943 to December 2003). Nevertheless, based on our past experience in growing white birch seedlings in greenhouses, it appears that the species is capable of acclimating to continuous warming to more than 40°C in the early afternoon on sunny summer days (see Figure 1). In this current study, we test the hypothesis that CO _2_ elevation will enhance the photosynthetic performance of white birch seedlings growing in a progressively warming environment.

## Materials and methods

### Plant materials

White birch ( *Betula papyrifera* Mash.) seedlings were grown from seeds in the greenhouses at the Thunder Bay campus of Lakehead University. The growing medium was a mixture of peat moss and vermiculite (1:1 (v/v)).

### Experiment design

The seedlings were subject to a progressive warming in the greenhouses as the season progressed from March to August ( Figure 1). The temperatures in all the greenhouses were monitored and recorded using a computerized environment control system ( *Argus*, Vancouver, Canada). The highest recorded temperature in the greenhouse was 44.8°C in the later stages of the experiment ( Figure 1). The seedlings were grown under two CO _2_ concentrations (i.e., the ambient (360 µmol mol ^-1^) and elevated (650 µmol mol ^-1^)). The two CO _2_ treatments were conducted simultaneously in separate greenhouses with identical design and dimensions. The CO _2_ elevation was achieved using *Argus* CO _2_ generators ( *Argus*, Vancouver, Canada). A photoperiod of 16-hour was maintained (the natural light was supplemented by high-pressure sodium lamps on cloudy days, early mornings and late evenings).

The moisture content of the growing medium was maintained at around 50%, as measured using a *HH2 Moisture Meter* ( *DELTA-T DEVICES*, Cambridge, UK). The seedlings were watered up to twice a day during the summer to maintain the soil moisture condition. The seedlings were fertilized weekly with a solution of 100 µmol mol ^-1^ N, 35 µmol mol ^-1^ P and 66 µmol mol ^-1^ K.

### Simultaneous measurements of *in situ* gas exchange and chlorophyll fluorescence

The foliage gas exchange was measured using a PP-Systems *CIRAS-1* open gas exchange system ( *Hitchin*, Hertfordshire, UK). The environmental conditions in the broad-leaf chamber were controlled automatically. The environmental conditions for measuring the Pn-C _i_ (C _i_ = intercellular CO _2_ concentration) curve were as follows: 26°C and 37°C air temperature, which were close to the highest temperatures in the early and late period of the experiment, 800 µmol m ^-2^s ^-1^ PAR (PAR = photosynthetically-active radiation) and 50% relative humidity. The *in vivo* maximal carboxylation rate (V _cmax_), PAR-saturated electron transport rate (J _max_), triose phosphate utilization (TPU) and other relevant parameters were calculated from the Pn-C _i_ curves according to Farquhar *et al.* ^26^, van Caemmerer and Farquhar ^27^, Sharkey ^28^, Harley and Sharkey ^29^ and Harley *et al.* ^30^. The Pn-C _i_ curves were fit using the *Photosyn Assistant* software ( *Dundee Scientific*, Scotland, UK) to estimate V _cmax_, J _max_ and TPU. The parameters for the kinetics of RuBiscCO, i.e., Kc, Ko and τ, and their temperature dependencies were adopted from Harley *et al.* ^30^ and Wullschleger ^31^.

Three seedlings were selected randomly from each treatment combination for the measurement. The measurement was taken on the top 5 ^th^ mature leaf. All the *in situ* measurements were made between 9:00 and 11:30 AM with the seedlings in their original positions and conditions of the treatments.

The chlorophyll fluorescence was measured using a *FMS-2* portable pulse-modulated fluorometer ( *Hansatech Instruments Ltd*. Norfolk, UK). The probe was integrated in the leaf chambers of the gas exchange system and the control software for the two systems was also integrated to allow the simultaneous measurement of gas exchange and chlorophyll fluorescence. The following variables were obtained: fluorescence intensity at any time, F; the maximal fluorescence in light, F _m_’; the actual photochemical efficiency of PSII in light, (F _m_’-F)/F _m_’ or ΔF/F _m_’, which is the efficiency under the actual degree of reaction centre closure ^32^. F _m_’ was obtained by illuminating the foliage with a pulse of strong light (around 14000 µmol photons m ^-2^s ^-1^) for 800 ms. The ΔF/F _m_’ was measured simultaneously with each gas exchange measurement. Both gas exchange and chlorophyll fluorescence were measured after 5 months of the treatments.

The apparent rate of total electron transport (J _T_) and its partitioning between carboxylation (J _c_) and oxygenation (J _o_) were calculated based on the methods of Farquhar *et al.* ^26^, Genty *et al.* ^33^ and Epron *et al* ^34^.

### Statistical analysis

All the data were examined graphically for the normality of distribution (probability plots for residual analysis) and the homogeneity of variance (scatter plots) using the Data Desk (version 6.01, Data Description, Inc. 1996) ^35^ before the Analysis of Variance (ANOVA) was carried out. Some of the data were log-transformed to meet the two assumptions for ANOVA. The data were analyzed using the two-way ANOVA procedure of the Data Desk. When the interaction between temperature and CO _2_ was significant, Scheffe’s F test for post hoc pairwise comparisons was conducted.

## Results

### *In situ* photosynthetic gas exchange

**Figure 2.**
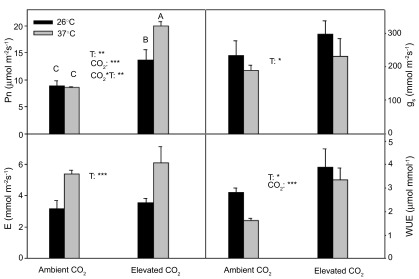
Pn, *g*_s_, *E* and WUE (mean ± SD, n=3–4) for current year white birch seedlings after they were exposed to continuous warming under ambient CO _2_ and elevated CO _2_ concentrations for 5 months. The *in situ* measurements were taken at 26°C and 37°C under ambient CO _2_ and elevated CO _2_. The significance levels (*** = *P*<0.001, ** = *P*<0.01, * = *P*<0.05). If the interaction between measurement temperature and CO _2_ was significant for a given parameter, Scheffe’s F test for post hoc pairwise comparisons was conducted. Means sharing the same letter or letters are not significantly different.

There was a significant ( *P*<0.01) interactive effect of temperature and CO _2_ on Pn ( Figure 2). Pn was higher ( *P*<0.01) at 37°C than at 26°C under elevated CO _2_( Figure 2), but there was no significant ( *P*>0.05) temperature effect on Pn under ambient CO _2_. CO _2_ elevation significantly increased Pn at both temperatures ( *P*<0.05, *P*<0.001 at 26°C and 37°C, respectively). *g*_s_ significantly ( *P*<0.05) decreased at 37°C under both ambient and elevated CO _2_( Figure 2), and there was no significant ( *P*>0.05) CO _2_ effect on *g*_s_. Meanwhile high temperature significantly ( *P*<0.05) stimulated *E* under both ambient and elevated CO _2_( Figure 2). Water-use efficiency (WUE) was significantly ( *P*<0.05) higher at 26°C than that at 37°C under both CO _2_ regimes. CO _2_ elevation greatly ( *P*<0.001) increased WUE at both temperatures.

High temperature significantly reduced *C _i_* under both ambient and elevated CO _2_ ( *P*<0.05, *P*<0.01, respectively) and, also, elevated CO _2_ significantly ( *P*<0.001) increased *C _i_* at both temperatures.

### *In vivo* RuBisCO activity

**Figure 3.**
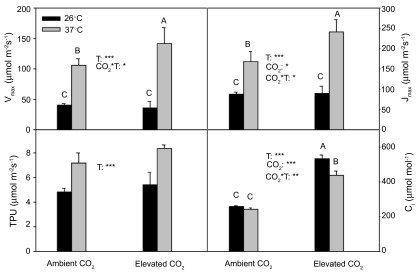
V _cmax_, J _max_, TPU and C _i_ in current year white birch seedlings. V _cmax_, J _max_ and TPU were derived from A-C _i_ curves, which were measured at 26°C and 37°C under ambient CO _2_ and elevated CO _2_. See Figure 2 for other explanations.

V _cmax_, J _max_ and TPU at 37°C were significantly ( *P*<0.001) higher than those at 26°C ( Figure 3). The temperature dependencies of V _cmax_ and J _max_ were changed by CO _2_, and those values at 37°C enhanced much more ( *P*<0.05) under elevated CO _2_ than under ambient CO _2_.

### Photosystem II efficiency and electron transport partitioning to carboxylation and oxygenation

**Figure 4.**
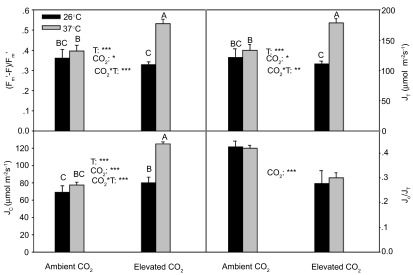
(F _m_’-F)/F _m_’, J _T_, J _c_ and J _o_/J _T_ in the current year white birch seedlings (F _m_’-F)/F _m_’ and J _T_ were derived from chlorophyll fluorescence measurements, and J _c_ and J _o_ were derived from both chlorophyll fluorescence and gas exchange measurements. See Figure 2 for other explanations.

There was a significant ( *P*<0.001) interactive effect of CO _2_ and temperature on (F _m_’-F)/F _m_’ and J _T_( Figure 4). (F _m_’-F)/F _m_’ and J _T_ greatly increased at 37°C as compared to at 26°C under elevated CO _2_, and there was no significant temperature effect on (F _m_’-F)/F _m_’ and J _T_ under ambient CO _2_.

The pattern of CO _2_ and temperature effects on J _c_ was almost the same as (F _m_’-F)/F _m_’ and J _T_( Figure 4), and J _c_ was greater ( *P*<0.001) at 37°C than that at 26°C under elevated CO _2_, and there was no significant temperature effect on J _c_ under ambient CO _2_. Elevated CO _2_ greatly suppressed J _o_/J _T_, and there was no significant (P > 0.05) effect of temperature on J _o_/J _T_( Figure 4).

A-Ci curves from gas exchange at 26-37°C CO2(out)=CO2 concentration in the ambient CO2(diff)=CO2 concentration difference between the ambient and leaf cuvette Light=photo flux density H2O(out)=water vapor pressure in the ambient H20(diff)=water vapor pressure difference between the ambient and leaf cuvette Cuvette temp.=temperature in the cuvette L area=leaf area Flow=flow rate Evap=transpiration rate Cond=stomatal conductance Leaf T=leaf temperature A=net photosynthetic rate Ci=intercellular CO2 concentration

PSII efficiency-CO2 curves Fs=stable fluorescence intensity Fm'=maximal fluorescence intensity in light (Fm'-Fs)/Fm'=actual PSII efficiency in light

Temperature data Air temperature against time

## Discussion

Our results suggest that the photosynthetic mechanisms of white birch seedlings have high capacity to acclimate to a progressively warming environment, particularly under elevated CO _2_. This result is in contrast to the results of most studies with a single step warming treatment. Larcher ^36^ has suggested that plants’ optimal temperature is closely related to the climate in which they grow. The measurement temperatures of 26°C and 37°C used in this study are believed to be the normal (or optimal) and stressful temperature, respectively, for most boreal forest tree species growing at their natural environments. Zhang *et al.* ^37^ have found that the Pn of mature oak even in warm-temperate zones decline greatly at temperatures over 30°C, as compared to measurements at temperatures of 20–30°C, which occurs naturally north of temperate zones or even warm-temperate zones. However, in this experiment the Pn of white birch didn’t decline at 37°C under ambient CO _2_, as compared to that at 26°C; furthermore, Pn increased substantially at 37°C under elevated CO _2_. These results indicate that the photosynthetic mechanisms of white birch acclimated to the progressive warming environment, and this high temperature acclimation was greatly strengthened by elevated CO _2_. Long ^20^ argued that CO _2_ elevation could change the photosynthesis dependence of temperature.

The activity of RuBisCO is highly temperature-dependent. According to Jordan and Ogren ^38^, the Rubisco’s specificity for CO _2_/O _2_ decreases as increasing temperatures over the optimal range, but the increase in RuBisCO oxygenation will exceed that of carboxylation because the solubility of CO _2_ declines faster than that of O _2_ at even higher temperatures, resulting in a decline in net photosynthetic rate. White birch’s acclimation to warming was also evidenced by V _cmax_ measured at the two different temperatures and two CO _2_ regimes. V _cmax_ at 37°C was much higher than at 26°C under both ambient and elevated CO _2_, indicating a shift in the temperature dependency of RuBisCO. Furthermore, the partitioning of total electron transport to oxygenation was not significantly different between the two temperatures under either ambient CO _2_ or elevated CO _2_, suggesting that the higher temperature did not change the RUBisCO specificity for CO _2_/O _2_ which could be a contributing factor for the enhanced acclimation of photosynthesis to the progressive warming. Overdieck *et al.* ^15^ have also found that both the temperature treatment alone and the combination of elevated CO _2_ and temperature depressed V _cmax_ in Scots pine at temperatures below the optimum range, but increased V _cmax_ when the temperature was above the optimum. Additionally, the magnitude of the change in V _cmax_ increased as temperature increased.

The decrease in C _i_ at the high temperature could be attributable to either enhanced RuBisCO activity or declines in stomatal conductance or both. Not only V _cmax_, but J _max_ and TPU were also higher at 37°C than at 26°C, suggesting that the CO _2_ assimilation process, including carboxylation, electron transport for RuBP regeneration, ATP supply and the translocation of the primary photosynthates, all maintained at high levels in the warm environment. In this study, there was no down-regulation of RuBisCO activity in association with the CO _2_ elevation, to the contrary, CO _2_ elevation greatly increased V _cmax_ and J _max_ at 37°C, as well as Pn at both 26°C and 37°C.

While high temperature enhanced V _cmax_ under both ambient and elevated CO _2_, the increases in actual PSII efficiency (ΔF’/F _m_’) and J _c_ associated with the high temperature only occurred under elevated CO _2_, suggesting that the high temperature did not significantly affect the total electron transport, and its partitioning to carboxylation, t under the ambient CO _2_. Conversely, the partitioning of total electron flow to oxygenation increased more than 40% in response to the high temperature under elevated CO _2_. The reduced electron transport partitioning to carboxylation and low C _i_ might explain why Pn was relatively low at 37°C under the ambient CO _2_, even though the corresponding V _cmax_ was quite high, implying that the slow electron transport to carboxylation and CO _2_ supply at high temperature under ambient CO _2_ didn’t match the high activity of RuBisCO. This again confirms Kirschbaum’s theoretical analysis that photosynthesis has a higher potential to be stimulated by CO _2_ elevation at high temperatures than at low temperatures ^16^.
