# Supplementary figures and images for: CO 2 elevation improves photosynthetic performance in progressive warming environment in white birch seedlings
Source: F1000Res. 2013 Jan 15;2:13. [Version 1] doi: 10.12688/f1000research.2-13.v1 (PMC3869490; doi:10.12688/f1000research.2-13.v1)

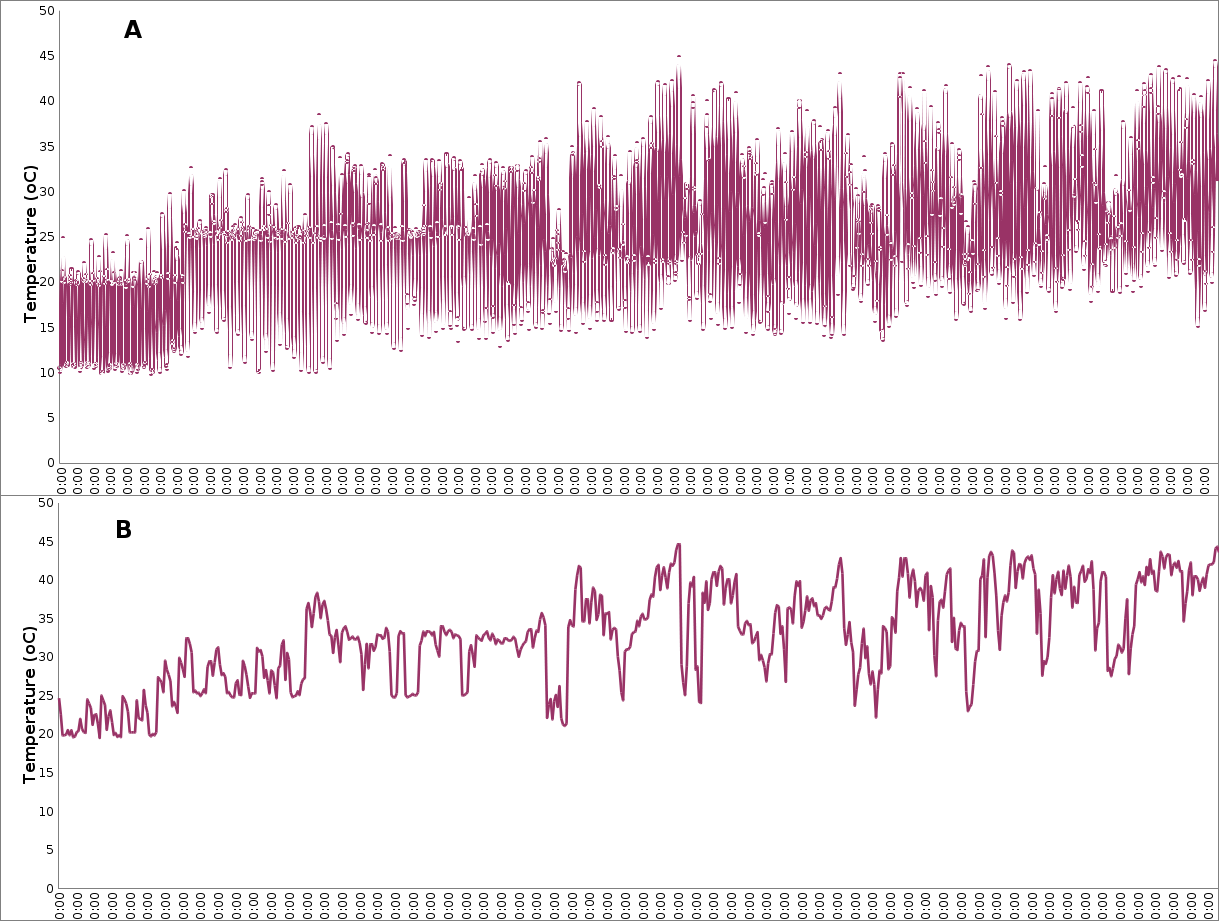

Supplement: Temperature data — Air temperature against time [file f1000research-2-1105-s0000.tgz › temperature_data.png]
