# Supplementary material for: CO 2 elevation improves photosynthetic performance in progressive warming environment in white birch seedlings
Source: F1000Res. 2013 Jan 15;2:13. [Version 1] doi: 10.12688/f1000research.2-13.v1 (PMC3869490; doi:10.12688/f1000research.2-13.v1)
Supplement: Peer review history

# Peer review: Referee response for version 1

**Published:** 2013-07-01

**Authors:** Christian Koerner

**Affiliations:** 1 Institute of Botany, University of Basel, Basel, CH -4056, Switzerland

Having stated that the work makes a very sound impression to me and reflects state of the art methodology, I still have concerns about the implied meaning. The authors carefully avoid making any statement with regard to growth or productivity implications. However, why would one make such measurements if not for the implied meaning for growth and overall performance? From all what we know to date, such a link has not been shown outside horticultural conditions. It will be most unlikely that a birch seedling in the wild is carbon limited. Whatever the photosynthetic performance, such a seedling would only incorporate structural carbon to the extent nutrients permit, and these are finite per unit land area (except for N) and are competitively foraged for.

I have read this submission. I believe that I have an appropriate level of expertise to confirm that it is of an acceptable scientific standard.

# Response

**Published:** 2013-07-03

**Authors:** Shouren Zhang

**Affiliations:** Chinese Academy of Sciences, China

We appreciate the invaluable comments from Professsor Christian Koerner. The primary focus of this study was to investigate the physiological plasticity of white birch in response to gradually warming environmental conditions under the elevated CO _2 _conditions in the future, not growth. Growth is more closely related to the carbon balance and resource availability than to photosynthesis or any single physiological process. While short term physiological studies such as ours are important for understanding the physiological mechanisms and ability in plant responses to stressful environmental conditions, extrapolating the results to predict the growth performance of trees in the field is risky and not reliable because of the long life of trees and the dynamic nature of natural environmental conditions. Therefore, we have refrained ourselves from making such extrapolations.

# Peer review: Referee response for version 1

**Published:** 2013-02-08

**Authors:** Xianzhong Wang

**Affiliations:** 1 Department of Biology, Indiana University-Purdue, Indianapolis, IN, USA

This is certainly a valuable manuscript.

I have read this submission. I believe that I have an appropriate level of expertise to confirm that it is of an acceptable scientific standard.
